# Identification of Host Bloodmeal Source in *Ornithodoros turicata* Dugès (Ixodida: Argasidae) Using DNA-Based and Stable Isotope-Based Techniques

**DOI:** 10.3389/fvets.2021.620441

**Published:** 2021-02-17

**Authors:** Hee J. Kim, Gabriel L. Hamer, Sarah A. Hamer, Job E. Lopez, Pete D. Teel

**Affiliations:** ^1^Department of Entomology, Texas A&M AgriLife Research, College Station, TX, United States; ^2^Department of Veterinary Integrative Biosciences, College of Veterinary Medicine and Biomedical Sciences, Texas A&M University, College Station, TX, United States; ^3^Department of Pediatrics, Center for Tropical Medicine, Baylor College of Medicine, Houston, TX, United States

**Keywords:** *Ornithodoros turicata*, bloodmeal analysis, stable isotope, soft tick, DNA-based technique

## Abstract

The ecology and host feeding patterns of many soft ticks (Ixodida: Argasidae) remain poorly understood. To address soft tick–host feeding associations, we fed *Ornithodoros turicata* Dugès on multiple host species and evaluated quantitative PCR (qPCR) and stable isotope analyses to identify the vertebrate species used for the bloodmeal. The results showed that a qPCR with host-specific probes for the *cytochrome b* gene successfully identified bloodmeals from chicken (*Gallus gallus* L.), goat (*Capra aegagrus hircus* L), and swine (*Sus scrofa domesticus*) beyond 330 days post-feeding and through multiple molting. Also, qPCR-based bloodmeal analyses could detect multiple host species within individual ticks that fed upon more than one species. The stable isotope bloodmeal analyses were based on variation in the natural abundance of carbon (^13^C/^12^C) and nitrogen (^15^N/^14^N) isotopes in ticks fed on different hosts. When compared to reference isotope signatures, this method discerned unique δ^13^C and δ^15^N signatures in the ticks fed on each host taxa yet could not discern multiple host species from *O. turicata* that fed on more than one host species. Given the significance of soft tick-borne zoonoses and animal diseases, elucidating host feeding patterns from field-collected ticks using these methods may provide insight for an ecological basis to disease management.

## Introduction

The identification of host bloodmeal sources in arthropod vectors provides vital information in vector-borne disease ecology, which enables vector-specific control and a proactive vector–host–pathogen risk assessment (1–7). Numerous bloodmeal analyses have evolved over time. Serological methods, such as the precipitin test, which rely on antibody reaction to host blood date back to the 1940s (8, 9), and the advent of polymerase chain reactions (PCRs) and quantitative PCR (qPCR) have enabled a suite of DNA-based arthropod bloodmeal analysis techniques to be developed (10). DNA-based techniques for the bloodmeal analysis have been used successfully for a wide variety of arthropod vectors, including mosquitoes, kissing bugs, and black flies (10). However, some arthropod vectors with a prolonged generation time, starvation periods, and molting between life stages pose challenges to the DNA-based bloodmeal analyses.

The primary challenge of conducting bloodmeal analyses in arthropod vectors enduring starvation periods lasting months to years, such as in ticks, is that the DNA obtained from previous life stage bloodmeals is degraded during molting (11, 12). To overcome this challenge, techniques such as reverse line-blot hybridization have shown potential due to its improved sensitivity to low quantities of DNA and the ability to use a large panel of host-specific probes (13, 14). However, this approach to bloodmeal analysis may be limited by availability of existing host blood probe data and optimization procedures (10, 13, 14).

The application of stable isotopes (SIs) to tick bloodmeal analyses offers the advantage of not needing to rely on host blood DNA detection. SI analyses determine the relative ratios of heavy to lighter elements within the organism that are influenced by its diet (3, 15–18). Proof-of-concept studies indicate that SI ratios of carbon and nitrogen from host bloodmeals are detectable in hard ticks after feeding on known hosts and molting (19), with the ability to discern among host taxa for up to 34 weeks post-molt in *Amblyomma americanum* (L.) (7). However, differentiation between ticks fed on ecologically similar hosts (*Peromyscus leucopus* mice vs. *Tamias striatus* chipmunks) was not possible, suggesting that SI analyses for bloodmeal host detection may be useful in sorting ticks to the level of the feeding guild, but not to the host species (20). Further, SI analyses require different vertebrate hosts' unique SI profiles to interpret the results from arthropods (15, 18, 21).

Bloodmeal analysis studies for argasid ticks are relatively rare (22–27) compared to those conducted for ixodid species. Some argasid ticks, such as *Ornithodoros turicata* Dugès (Ixodida: Argasidae), are known to survive for years without a bloodmeal (28, 29). The medical–veterinary importance of *O. turicata* and the ease with which it can be reared and maintained in the laboratory make it a good model for evaluating DNA- and SI-based tools for host bloodmeal identification.

*O. turicata* is a well-established vector and reservoir of *Borrelia turicatae*, one of the spirochetes that can cause tick-borne relapsing fever (TBRF) (30–36) and a potential vector of the African swine fever virus (37, 38). Collection records indicate that the distribution of *O. turicata* ranges from southern Mexico to the southwestern United States, as well as the state of Florida (39–41). Despite its early description in 1876 and subsequent studies linking its vector potential to TBRF as early as the 1930s, the vector ecology and host preferences of *O. turicata* remain relatively unstudied. This knowledge gap may be a result of biological and behavioral attributes of *O. turicata* that pose challenges to conducting surveillance in its native environment. For example, *O. turicata* is generally considered a nocturnal organism with an affinity toward microhabitats found in caves, burrows, nests, and cavities with host activity and is seldom found in relatively accessible open environments (39, 42–44). It is a generalist with a broad host range including taxa of mammals, birds, and reptiles; has up to seven nymphal instars; and may require one or more bloodmeals at each life stage before molting to the next (40, 43). With each host feeding event lasting just a few minutes (45), *O. turicata* is rarely found attached to its host. Therefore, current survey methods for *O. turicata* and other argasid ticks are limited to labor-intensive and time-consuming techniques such as the CO_2_ baiting, debris-filtering methods (40, 46), and animal burrow vacuuming techniques (47).

The objective of this study is to compare DNA- and SI-based bloodmeal analyses on colonized *O. turicata* with known bloodmeals on vertebrates at different days post-feeding. The development of reliable techniques to identify host bloodmeals would provide valuable methods and applications to ecological studies and surveillance programs.

## Materials and Methods

### Tick (*O. turicata*) Colony

Adults and late-instar nymphs of *O. turicata* used in this study were obtained from a colony maintained at the Tick Research Laboratory, Texas A&M AgriLife Research, College Station, TX, United States. The colony originated from specimens collected in a natural cavern in Travis County, TX, United States in 1992. The *O. turicata* colony has been maintained under a 14:10 (light: dark) photoperiod, 25.0 ± 3.0°C, and 80–85% relative humidity and fed approximately once a year using young cockerels (*Gallus gallus* L.) as bloodmeal hosts according to procedures approved by the Institutional Animal Care and Use Committee of Texas A&M University (AUP no. 2014-255).

### Cohort Preparation

Timelines and protocol overview are outlined in Table 1. Four *O. turicata* cohorts, each consisting of approximately 600 larvae, were prepared by transferring 30 larvae from the progeny of each of 20 female ticks using a camel-hair brush. The cohorts were reared to the fourth-instar nymph stage using different combinations of chicken (*G. gallus* L.), goat (*Capra aegagrus hircus* L.), and swine (*Sus scrofa domesticus*) blood. The first group was labeled “EC,” short for “Exclusively fed on Chicken,” and was reared exclusively on live chickens for four bloodmeals. The second group was labeled “CG,” short for fed on “Chicken and Goat,” and was reared on live chickens for three bloodmeals and a final bloodmeal on commercially acquired, mechanically defibrinated goat blood (Rockland Immunochemicals Inc., Limerick, PA, United States) via an artificial membrane protocol developed by Kim et al. (48). The third group was labeled “CS,” short for fed on “Chicken and Swine,” and was reared on live chickens for three bloodmeals and final bloodmeal on commercially acquired, mechanically defibrinated swine blood (Rockland Immunochemicals Inc., Limerick, PA, United States) via an artificial membrane. The fourth group was labeled “ES,” short for “Exclusively fed on Swine,” and was reared solely on commercially acquired, mechanically defibrinated swine blood via an artificial membrane. At 30-day intervals after their final bloodmeal (up to 9 months), a group of 10 ticks was harvested from each cohort, with five ticks serving as replicates for DNA-based analysis and five ticks serving as replicates for SI-based bloodmeal analysis shown in Table 1.

**Table 1:** Timeline for the development and sampling scheme of four experimental *Ornithodoros turicata* cohorts fed on different kinds of host blood.

**Experiment (days)**	***O. turicata* (state)**	**Note**
-	Larvae to 2N	EC, CG, and CS cohorts reared to 2N using chicken blood. ES cohort reared to 2N using swine blood
0	2N (engorged)	EC, CG, and CS cohorts fed on chicken blood. ES cohort fed on swine blood
30	3N (unfed)	Samples collected for qPCR and SI analyses from each cohort
60	3N (engorged)	Final bloodmeal for all cohort EC cohort fed on chicken blood CG fed on goat blood CS fed on swine blood SS fed on swine blood Samples collected for qPCR and SI analyses from each cohort
90	4N 0M (freshly molted)	Samples collected for qPCR and SI analyses from each cohort
120	4N 1M (1 month post molt)	Samples collected for qPCR and SI analyses from each cohort
150	4N 2M (2 months post molt)	Samples collected for qPCR and SI analyses from each cohort
180	4N 3M (3 months post molt)	Samples collected for qPCR and SI analyses from each cohort
210	4N 4M (4 months post molt)	Samples collected for qPCR and SI analyses from each cohort
240	4N 5M (5 months post molt)	Samples collected for qPCR and SI analyses from each cohort
270	4N 6M (6 months post molt)	Samples collected for qPCR and SI analyses from each cohort
330	4N 7M (9 months post molt)	Samples collected for qPCR and SI analyses from each cohort

*All O. turicata cohorts were maintained under a 14:10 (light: dark) photoperiod, 25.0 ± 3.0°C, and 80–85% relative humidity*.

*N, instar nymph; M, month*.

### DNA Extraction and qPCR Analysis

Ticks from each sample group (*n* = 5) outlined in Table 1 were used for DNA extraction and qPCR analysis. In summary, five ticks from each cohort (EC, CG, CS, and ES) were collected immediately after their last bloodmeal, after a molt (~30 days post last bloodmeal), for six consecutive months (~4-week interval), and at 9 months post-molt. Surface contaminants on each tick were removed by briefly placing the tick in a 50% bleach solution for 30 s and then washing with water as outlined by Graham et al. (49). The whole-body DNA extraction was performed per the manufacturer's instructions using the E.Z.N.A.® Tissue DNA Kit (Omega Bio-Tek, Norcross, GA, Unite States) with overnight lysis. DNA was extracted from each *O. turicata* by cutting it into two equal segments in a sterile centrifuge tube, exposing its midgut content to a lysis buffer solution with a final elution volume of 50 μl. DNA from aliquots of each host blood (15 μl per each host blood) used to feed *O. turicata* treatment groups for this study was also extracted to serve as positive controls for their respective treatment groups. Both water-template and no-template wells served as negative controls.

The qPCR analysis was performed to detect the *cytochrome b* (*cytb*) gene in the extracted DNAs using host blood-specific primers and probes, as previously described by Cupp et al. (50). The *cytb* gene was selected as the appropriate molecular marker for the bloodmeal analyses because the primers are vertebrate-specific and would not amplify *O. turicata* DNA (50). Primer and probe sequences used for qPCR are listed in Table 2. Moreover, each probe was tagged with a unique reporter dye to aid in differentiating between vertebrate hosts. The unmodified *cytb* primers and probes for chicken and goat were used as described by Woods et al. (51). The primer and probe for the swine blood designed in this study used the Beacon Designer 8.0 software (Premier Biosoft, Palo Alto, CA, United States) based on *Sus scrofa* mitochondrion genome (GenBank accession #AF034253.1).

**Table 2:** Primer and probe sequences for host-specific *cytb* gene used as a molecular marker in qPCR assays to identify host blood source in colony-reared *Ornithodoros turicata*.

**Host blood**	**Gene**	**Amplicon size**	**Primer and probe sequences**	**References/GenBank accession number**
Chicken	*cytb*	162	Forward primer 5′-CCTCTACAAGGAAACCTCAAACAC-3′	(51)
			Reverse primer 5′-GACTAGGGTGTGTCCAATGTAGG-3′	
			Probe 5′-ROX-CGCCATAGTCCACCTGCTCTTCCTCCA-BHQ-3′	
Goat	*cytb*	125	Forward primer 5′-TCCTCCCATTCATCATCACAGC-3′	(51)
			Reverse primer 5′-TGGTGTAGTAAGGGTGAAATGGG-3′	
			Probe 5′-ROX-CGCCATAGTCCACCTGCTCTTCCTCCA-BHQ-3′	
Swine	*cytb*	176	Forward primer 5′-CTACGGTCATCACAAATCTACTATCAG-3′	This study/AF034253.1
			Reverse primer 5′-GTGCAGGAATAGGAGATGTACG′	
			Probe 5′-Cy5-ATCGGAACAGACCTCGTAGAATGAATC-BHQ-3′	

The LightCycler® 96 system (Roche Diagnostics Corporation., Indianapolis, IN, United States) was used for all qPCR analyses with the following conditions: initial denaturation at 95°C for 10 min, followed by 40 cycles at 95°C for 30 s and 60°C for 1 min. Subsequently, nuclease-free water and whole-blood DNA extracts were used as the qPCR negative and positive controls, respectively. Each qPCR assay used a 25-μl total reaction volume that had 400 nM of forward and reverse primers each, 200 nM of probe, and 4.5 μl of DNA template (concentration unknown) using the Bio-Rad iTaq Universal Master Mix (Bio-Rad, Hercules, CA, United States). A total of 750 qPCR assays were conducted on DNA extracts. DNA extracted from host blood (chicken: 211.7 ng/μl, swine: 9.26 ng/μl, or goat: 17.4 ng/μl) was used as positive controls in the assays. Among the 750 assays, 365 qPCR assays were labeled as “unmatched samples,” which denoted DNA extracts from *O. turicata* sample groups tested using host-specific primer and probes of blood that was not used to rear them. This was done to further assess any cross-reactivity across host blood. The remaining 385 qPCR assays were labeled as “matched samples,” denoting DNA extracts from *O. turicata* sample groups tested using host-specific primers and probes of blood used by the corresponding cohort. The matched samples included the ticks reared on two host species when the primer–probe set was specific to one of the hosts. The qPCR results were interpreted as positive when a DNA sample cycle threshold (Ct) value was <35. This threshold value was determined conservatively based on preliminary trials that showed that late and non-specific amplification of negative controls occurred at or above 36 cycles.

### SI Analysis

SI analysis was conducted using *O. turicata* from each tick sample group (*n* = 5) collected concurrently as those gathered for qPCR. Elemental analysis isotope ratio mass spectrometry (EA-IRMS) at the Stable Isotope Geosciences Facility at Texas A&M University, College Station, TX, United States, was used to analyze individual ticks for carbon (^13^C/^12^C) and nitrogen (^15^N/^14^N) isotopic values as described by Hamer et al. (7). The EA combusted the tick and blood samples at 1,200°C, separating CO_2_ and N_2_ gases, and analyzed on the EA-IRMS. The standard delta (δ) notation δX = [(Rsample/Rstandard) – 1] × 1,000, where R was the ratio of the heavy to light SI in the sample and standard, was used to represent the results. Next, results were referenced according to the Vienna Pee Dee Belemnite (VPDB) carbonate standard for δ^13^C and relative to air for δ^15^N. Finally, the range of δ^13^C and δ^15^N values of samples for a 2-point calibration and internal laboratory standards every ~12 unknowns was used to measure analytical precision as described by Hamer et al. (7). Samples from each host blood type used to feed corresponding cohorts were used to generate reference isotope signatures in the SI analysis.

### Statistical Data Analysis

The statistical program JMP® Pro 12 (SAS Co., Cary, NC, United States) was used for all statistical analyses. The exact Cochran–Armitage trend test was conducted to assess any difference in qPCR assay results of each *O. turicata* sample group (*n* = 5) based on the experiment days (length of starvation). A chi-square test was conducted to determine the associations between each tick's host feeding history (EC, CG, CS, and ES) and the qPCR results (positive or negative for each assay). Pillai's trace multivariate analysis of variance (MANOVA) was used to compare δ^13^C and δ^15^N values of host blood, all unfed third-instar nymphs, all engorged third-instar nymphs, sample groups from each cohort, and combined sample group values for all cohorts. When MANOVA indicated a significant difference, a *post-hoc* test using Tukey's honestly significant differences was conducted to assess pairwise differences in both δ^13^C and δ^15^N based on an alpha level of 0.05.

## Results

### qPCR Analysis

All 365 “unmatched samples” tested negative in the qPCR, which denotes no significant cross-reactions (*P* < 0.0001, CI = 0, 0.01) among host *cytb* genes and non-corresponding host primers and probes. The chicken *cytb* gene was detected in at least one replicate within each five-tick sample group fed on chicken blood in all EC, CG, and CS cohorts across the entire experiment period (Table 3). The *O. turicata* samples from the EC cohort had the highest overall qPCR-positive prevalence of 98%, followed by the samples from the CG cohort with 76% and the samples from the CS group with 60%. There were no significant differences in qPCR results of *O. turicata* samples based on the experiment day (length of starvation) in the EC cohort (*P* = 0.10, Cochran–Armitage trend test) and CG cohort (*P* = 0.35, Cochran–Armitage trend test). However, a significant difference was observed in the qPCR array results in tick samples from the CS cohort based on experiment days (*P* < 0.01, Cochran–Armitage trend test) (Table 3), indicating that the starvation period had affected the qPCR outcome.

**Table 3:** Summary of qPCR assays (Ct value < 35) based on experiment days per *Ornithodoros turicata* tick sample group (*n* = 5) fed on chicken (*Gallus gallus*), goat (*Capra aegagrus hircus*), and swine (*Sus scrofa domesticus*) blood.

***O. turicata* state**	**Experiment days**	**EC**	**CG**	**CS**	**CG**	**CS**	**ES**
		**qPCR positives out of 5 samples (%) using chicken** ***cytb*** **primer and probe**			**qPCR positives out of 5 samples (%) using goat *cytb* primer and probe**	**qPCR positives out of 5 samples (%) using swine** ***cytb*** **primer and probe**	
2N (engorged)	0	5 (100)	4 (80)	5 (100)	0 (0)^*^	0 (0)^**^	5 (100)
F 3N (engorged)	60	5 (100)	2 (40)	5 (100)	5 (100)	5 (100)	5 (100)
4N 0M (freshly molted)	90	5 (100)	4 (80)	4 (80)	4 (80)	5 (100)	3 (60)
4N 1M (1 month post molt)	120	5 (100)	5 (100)	2 (40)	2 (40)	4 (80)	5 (100)
4N 2M (2 months post molt)	150	5 (100)	4 (80)	3 (60)	2 (40)	4 (80)	3 (60)
4N 3M (3 months post molt)	180	5 (100)	5 (100)	3 (60)	2 (40)	5 (100)	3 (60)
4N 4M (4 months post molt)	210	5 (100)	2 (40)	1 (20)	2 (40)	1 (20)	5 (100)
4N 5M (5 months post molt)	240	5 (100)	4 (80)	1 (20)	5 (100)	3 (60)	5 (100)
4N 6M (6 months post molt)	270	5 (100)	3 (60)	4 (80)	1 (20)	5 (100)	2 (40)
4N 9M (9 months post molt)	330	4 (80)	5 (100)	2 (40)	5 (100)	2 (40)	5 (100)
Range of % positive per cohort		80–100	40–100	20–100	20–100	20–100	40–100
Mean % positive per cohort		98	76	60	64.44	75.56	82.00
SD per cohort		6.32	22.71	29.81	31.27	29.63	23.94
Mean % positive across cohorts		71			-	75.71	
SD per all cohort		29.68			-	28.99	
Exact Cochran–Armitage trend test		*P* = 0.1	*P* = 0.35	*P* < 0.01	*P* = 0.38	*P* < 0.01	*P* = 0.27

*N, instar nymph; EC, exclusively fed on chicken blood; CG, fed on chicken and goat blood; CS, fed on chicken and swine blood; ES, exclusively fed on swine blood*.

* *2N CG cohort not fed on goat blood and excluded from statics analysis*.

** *2N CS cohort not fed on swine blood and excluded from statistical analysis*.

The goat *cytb* gene was detected in at least one replicate within each five-tick sample group fed on goat blood in the CG cohort during the entire experiment period (Table 3). The overall average qPCR-positive prevalence for goat DNA in *O. turicata* samples from the CG cohort was 64.4%. There were no significant differences in the qPCR assay results of *O. turicata* samples based on the length of starvation in the CG cohort (*P* = 0.38, Cochran–Armitage trend test) (Table 3). The swine *cytb* gene was detected in at least one replicate within each five-tick sample group fed on swine blood in both CS and ES cohorts during the entire experiment period (Table 3). The *O. turicata* samples from the ES cohort had a higher overall average qPCR-positive prevalence of 82.0%, followed by the samples from the CS cohort with 75.6%. There were no significant differences in the qPCR assay results of *O. turicata* samples based on the length of starvation in the ES cohort (*P* = 0.27, Cochran–Armitage trend test). However, a significant difference was observed in the tick samples from the CS cohort (*P* < 0.01, Cochran–Armitage trend test) (Table 3). Finally, there were no differences between the qPCR results of the tick samples based on the host blood (*X*^2^ = 2.33, df = 2, *P* = 0.31) (Table 4), indicating that the host blood did not affect the qPCR outcome.

**Table 4:** Summary of qPCR assay results based on host blood-specific primers and probes.

***Ornithodoros turicata* cohort**	**Primer–probe**	**Positive (%)**	**Total samples**
Fed on chicken blood	Chicken	142 (71.0)	200
Fed on goat blood	Goat	29 (64.4)	45
Fed on swine blood	Swine	106 (75.7)	140
		277 (71.9)	385
	*n*		385
	Chi-square df		2
	Chi-square value		2.33
	Chi-square *P*		*P* = 0.31

### SI Analysis

**Figure 1:**
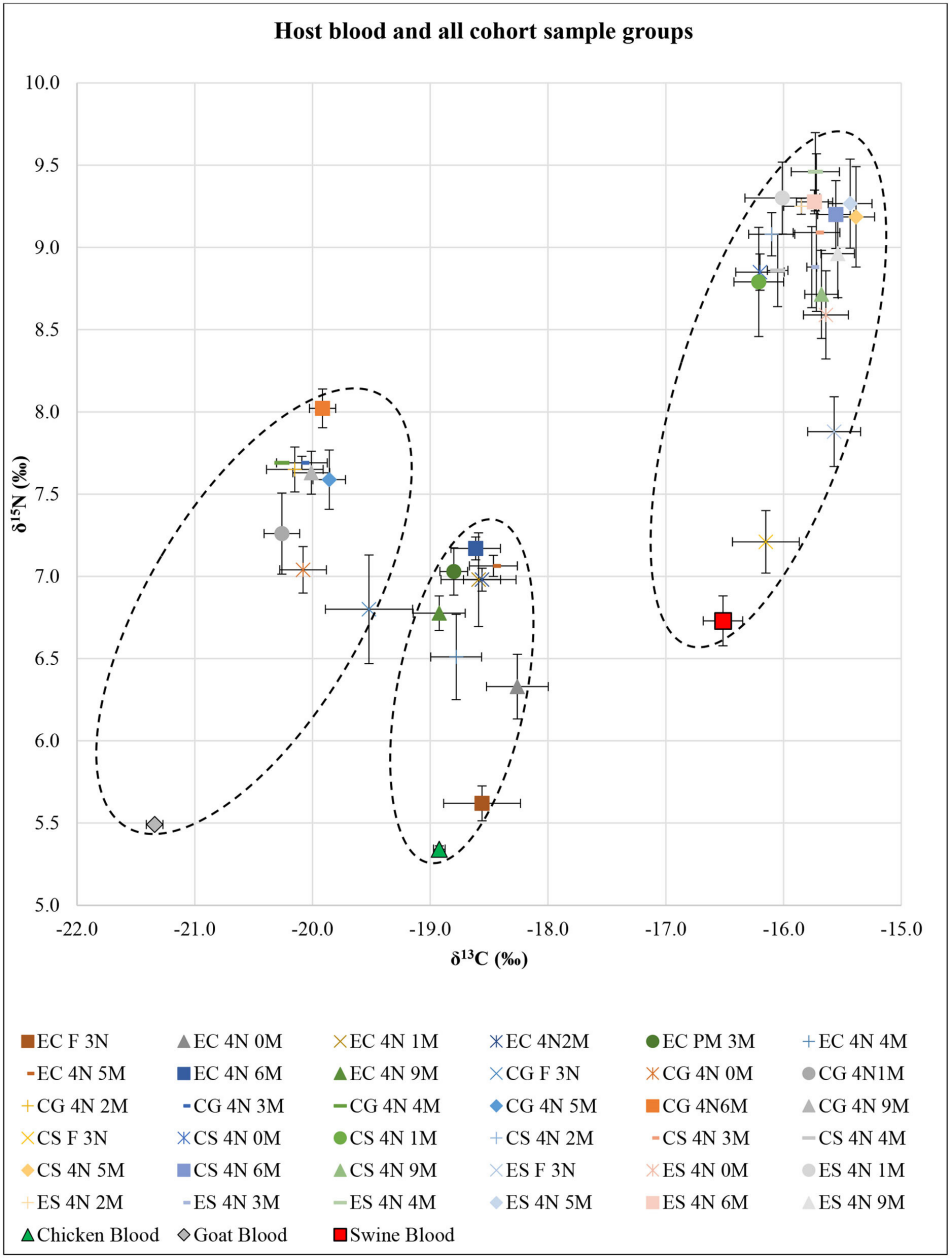
Isotopic results of all *Ornithodoros turicata* cohort sample groups based on post-molt time (*n* = 5 each) represented as δ^13^C and δ^15^N superimposed over the host blood results. X- and Y-axis error bars represent SEs around means. Dotted oval shapes encircle δ^13^C and δ^15^N values for each of the EC, CG, and CS+ES cohorts. EC, exclusively fed on chicken blood; CG, fed on chicken and goat blood; CS, fed on chicken and swine blood; ES, exclusively fed on swine blood; F 3N, third-instar nymph immediately after feeding; 4N 0M, fourth-instar nymph immediately after a molt; 4N 1M−4N 9M, fourth-instar nymph 1 month post-molt to fourth-instar nymph 9 months post-molt.

SI analysis results of δ^13^C and δ^15^N for each host blood were significantly different (*F* = 57.20; df = 4, 24; *P* < 0.01) (Figure 1). The *post-hoc* tests showed significant differences in δ^13^C for all pairwise combinations (*P* < 0.01 each), in which swine had the highest δ^13^C and goat had the lowest δ^13^C. The *post-hoc* test showed δ^15^N in swine blood was significantly higher than that in goat and chicken (*P* < 0.01 each).

SI analysis results of δ^13^C and δ^15^N for unfed third-instar *O. turicata* nymph samples from each cohort showed significant differences (*F* = 4.65; df = 6, 32; *P* < 0.01) (Figure 1). The *post-hoc* tests showed no significant differences in either δ^13^C or δ^15^N between EC, CG, and CS cohorts. On the other hand, the *post-hoc* tests for δ^13^C and δ^15^N showed that the ES cohort was significantly different from the EC, CG, and CS cohorts (*P* < 0.01 each). Similarly, in engorged third-instar *O. turicata* nymph samples, there were significant differences among the cohorts in δ^13^C and δ^15^N (*F* = 29.46; df = 6, 32; *P* < 0.01) (Figure 1). The *post-hoc* tests for δ^13^C and δ^15^N showed significant differences (*P* < 0.01 each), except between ES and CS cohorts (*P* = 0.55).

SI analysis results of δ^13^C and δ^15^N for EC, CG, CS, and ES fourth-instar *O. turicata* nymph samples based on the time since starvation showed significant differences among sample groups [(*F* = 3.04; df = 16, 72; *P* < 0.01) for EC, (*F* = 3.10; df = 16, 72; *P* < 0.01) for CG, (*F* = 3.91; df = 16, 72; *P* < 0.01) for CS, and (*F* = 3.22; df = 16, 72; *P* < 0.01) for ES] (Figure 1). The *post-hoc* test for δ^13^C showed no significant differences between all cohort samples. However, the *post-hoc* test for δ^15^N generally indicated significant differences between engorged third-instar nymphs and all post-molt fourth-instar nymphs. There were significant differences between engorged third-instar nymphs and all post-molt fourth-instar nymphs (*P* < 0.01 each) in EC and CS, between engorged third-instar nymphs and 3, 4, and 6 months post-molt fourth-instar nymphs (*P* < 0.01 each) in CG, and between engorged third-instar nymphs and 1–6 and 9 months post-molt fourth-instar nymphs (*P* < 0.01 each) in ES.

The combined δ^13^C and δ^15^N SI values comparing all fourth-instar *O. turicata* nymph cohorts regardless of their post-feeding time (average SI value, *n* = 45 per cohort) showed significant differences among the cohorts (*F* = 117.46; df = 6, 352; *P* < 0.01) (Figure 1). The *post-hoc* tests for both δ^13^C and δ^15^N showed significant differences between cohorts (*P* < 0.01 each) except between CS and ES cohorts. There were three clusters of δ^13^C and δ^15^N values observed (shown by dotted oval shapes) that represented EC, CG, and CS+ES cohorts (Figure 1). An isotopic shift of increasing δ^13^C and δ^15^N with tick age was observed for all treatment groups.

## Discussion

This study reports the first in-depth bloodmeal analysis of an experimentally fed argasid tick, *O. turicata*, using DNA- and SI-based techniques. The DNA-based bloodmeal analysis in this study accurately differentiated host blood in *O. turicata* cohorts including those receiving bloodmeals from different vertebrate species. Unexpectedly, the host-specific *cytb* gene was detectable in at least some replicates of each study group across the entire experiment period of 330 days using the DNA-based technique. Similarly, the SI-based bloodmeal analysis technique was proven capable of discerning the difference among *O. turicata* cohorts which fed on different single vertebrate host blood taxa (chicken, goat, or swine). However, the SI-based bloodmeal analysis failed to discern the difference between cohorts that fed only on blood from a single host taxon (e.g., swine only) from cohorts that fed on blood from two host taxa when the latter included the same vertebrate host blood as the single-host-blood cohorts (e.g., chicken then swine vs. swine only).

The SI analysis generated distinctive δ^13^C and δ^15^N values for each host blood as well as for the *O. turicata* cohorts that fed on different kinds of host blood. While the SI values of the host blood did not overlap directly with *O. turicata* that fed on the same host blood, a previous study has shown that the isotopic discrimination (i.e., the tick-blood spacing) was invariable, and an increase in nitrogen composition in the tick is expected relative to the composition of the blood on which the tick fed, given the increase in the level of the food chain (19). Furthermore, we observed an isotopic shift (increasing δ^13^C and δ^15^N) with tick age, which has also been observed in prior studies (7, 19) and which complicates the utility of this approach for tick bloodmeal analysis. SI analyses may be limiting when a large number of vertebrate species are expected as bloodmeal hosts. However, SI bloodmeal analysis could be viable if, during field or laboratory experiments, the vertebrate host community has restricted species richness and if the time post-feeding (e.g., >1 year) exceeds the ability of DNA-based approaches. However, sample cost is a consideration. The inclusion of an additional SI, sulfur (^34^S), is now available given that many laboratories have the ^13^C, ^15^N, and ^34^S combined analysis that can be used on the sample. However, the cost for the dual ^13^C-and-^15^N analysis is $8.50 per sample at the UC-Davis Stable Isotope Facility, while the cost of the ^13^C, ^15^N, and ^34^S triplex is $73.00 (https://stableisotopefacility.ucdavis.edu/index.html).

Prolonged detectability of vertebrate host DNA within *O. turicata* suggests that the processing and storing of host blood in *O. turicata* may be considerably different between argasid and ixodid species. Hamer et al. (7) conducted host-specific qPCR-based bloodmeal analysis on *A. americanum* and reported that the assay began to fail to detect host-specific *cytb* as early as 42 days post-feeding in adult sample groups and rarely detected the host beyond 40 weeks post-feeding. There are no clear explanations for differences in *cytb* integrity observed between these studies. Nonetheless, exploring the bloodmeal digestion process between *A. americanum* and *O. turicata* may elucidate a plausible explanation.

Bloodmeal processing in both ixodid and argasid species is composed of three phases. Hemolysis takes place during the first phase, which occurs immediately upon feeding and lasts 2 to 15 days. The second phase, also called the “rapid” digestion, takes place in the midgut of ticks and can last from several weeks to 3 months. Finally, the third phase, also called the “slow” digestion, occurs mainly in the apical branches of the diverticula and can last for years (52). The difference between ixodid and argasid tick digestion is the third digestion phase. Bloodmeal digestion in ixodid ticks occurs uniformly, and the ingested bloodmeal is evenly stored and consumed at a steady rate in the midgut as well as in the diverticula (52). On the other hand, bloodmeal digestion in the third phase of argasid ticks occurs at a variable rate because a substantial amount of bloodmeal is stored in the peripheral regions of the midgut diverticula with no digestive activity (52). This slow and uneven digestion of the bloodmeal allows argasid ticks to endure starvation that could last for years, as observed in *O. turicata* (45). In this study, *O. turicata* samples from CG and CS cohorts were able to maintain a detectable level of chicken *cytb* gene throughout two molts and starvation periods exceeding 9 months (Table 3). Indeed, the rate of biochemical processes (i.e., no digestive activity) in the peripheral regions of the midgut diverticula can slow down the digestion of the bloodmeal, thus prolonging the overall bloodmeal consumption (52). Nevertheless, the physical capacity of the peripheral regions of the midgut diverticula that store a previous bloodmeal may also force subsequent and newly acquired bloodmeal to be kept in the medial regions of the midgut where active digestion occurs (52). This “blocking” of storage space by the previous bloodmeal may allow residual bloodmeal from earlier feedings to remain for the entire tick life span of *O. turicata*, enabling qPCR analysis to detect multiple host *cytb* genes. In contrast, the qPCR analysis was useful in simultaneously detecting two host-specific *cytb* genes correctly across all *O. turicata* fed on multiple kinds of host blood. However, this documentation was based on the detection of a chicken bloodmeal which was taken prior to the goat or swine bloodmeal in the mixed-species sample groups. We did not attempt the reverse order by feeding the ticks on goat or swine and then chicken bloodmeals, which is important to note given that the bird blood likely had more DNA than the mammal blood.

The detectability of multiple host bloodmeals within *O. turicata* that experimentally fed on two species varied depending on the bloodmeal analysis techniques employed. The SI technique could not be used to discern the difference between single-host and multihost blood-fed *O. turicata* cohorts. For example, there were no differences between the overall δ^13^C and δ^15^N values of the CS and ES cohorts (Figure 1). Moreover, engorged third-instar nymphs from CS and ES cohorts showed no significant difference in their δ^13^C and δ^15^N values (Figure 1), despite each cohort being fed different kinds of host blood previously. This further strengthens the argument that the last bloodmeal *O. turicata* acquired determines the outcome of the SI analysis. In contrast, the qPCR analysis was useful in simultaneously detecting two host-specific *cytb* genes correctly across all *O. turicata* cohorts fed on multiple host blood. However, the detection of DNA in the different *O. turicata* cohorts ranged between one of five ticks to five of five ticks, indicating variation in either the retention of the DNA in the ticks or variation in the methodology (DNA extraction or qPCR). For example, we used 4.5 μl of template DNA from all samples and did not adjust based on the quantity of DNA, which was likely variable. Additionally, quantifying sample DNA and running these qPCR reactions in replicates would likely have strengthened the results and should be considered in future studies.

The duration of starvation could influence the outcomes of each type of bloodmeal analysis technique. For example, qPCR results for the CS cohort using swine-specific primers and probes seem to be influenced by the duration of starvation (Table 3). There was no logical explanation for this since the qPCR results of other groups, such as the ES cohort, which were also reared using swine blood were not affected by the duration of starvation. Since there were only five tick replicates per group, increasing the number of replicates per sample group may reduce the inconsistency observed in qPCR results for future studies. However, no sample tick group failed to retain detectable DNA at any time during the entire experiment period. Thus, it is uncertain how much improvement in the reproducibility of qPCR results can be made by increasing the number of ticks per sample group.

Different amounts of DNA that exist in different kinds of host blood provide another plausible explanation for the apparent influence of the starvation period on the outcomes of each type of bloodmeal analysis technique. Chicken blood, which consists of both immature and mature nucleated erythrocytes (53), presumably had higher overall DNA concentration in ticks. In contrast, goat and swine blood (and other mammals) are known to have immature nucleated erythrocytes that become anucleated once matured, attributing to relatively low DNA extract yield (54). While this study did not examine the proportion of *cytb* gene within the total DNA extract of host blood, an inference can be made based on the ubiquitous presence of the *cytb* genes in vertebrate hosts as part of their mitochondria, in that the relative proportions of *cytb* gene in the chicken, goat, and swine blood would be similar to that of total DNA extract (50, 55–57). Therefore, the *O. turicata* CS cohort, which fed on swine blood once, may not have had the chance to acquire and maintain the adequate amount of swine *cytb* gene throughout the entire experiment period compared to the *O. turicata* ES cohort, which had four opportunities to feed on swine blood. Moreover, the fact that the host blood type had no effect on the overall qPCR results of any tick sample groups (Table 4) further denigrates the significance of different qPCR results seen in the CS cohort. Therefore, an argument can be made that the inconsistency observed in qPCR results based on the length of starvation seen in CS cohort may not be attributed to a single reason but due to combinations of low sample number, lower DNA extract yield in swine blood, and variable rate of bloodmeal digestion of *O. turicata*.

Starvation duration influenced the outcomes of SI analysis differently than that of qPCR analysis. First, patterns of increased δ^13^C and δ^15^N values in engorged third-instar nymphs in each cohort compared to their corresponding host blood were observed. This observation could be due to SI (e.g., nitrogen) being enriched (15, 17, 58). The increase of δ^13^C and δ^15^N values in engorged *A. americanum* was also previously observed (7). However, the SI analysis failed to provide conclusive evidence for the SI fractionation, which occurs due to nutrient stress such as starvation. Such physiological stresses cause nitrogen fractionation via changes in the rate of amino acid consumptions, uric acid formations, and secretions (17, 21). Indeed, Hamer et al. (7) also reported changes in δ^13^C over time in *A. americanum* fed on chicken; however, data from this study were inconclusive to make such inference. This may be due to the inconsistent digestion rate in *O. turicata* mentioned above.

The applicability and limitation of the bloodmeal analysis techniques used in this study must be carefully considered in the contexts of *O. turicata* biology and ecology. For example, the qPCR analysis will require a catalog of specific host genes, primers, and probes. In contrast, SI analysis will require blood SI signatures of the animals circulating in the *O. turicata* habitat in order to conduct bloodmeal analyses accurately. Furthermore, the longevity of *O. turicata* ticks may pose unique considerations for understanding the vertebrate hosts that are important for feeding ticks. The longevity of *A. americanum* is typically <3 years, and overlapping generations found in their population structure may rarely consist of more than two generations (59). In this case, tick host feeding patterns observed in the population may closely resemble the actual host utilization as the host population dynamic may not change drastically within the typical generation time. On the other hand, the longevity of *O. turicata* may be measured in decades (28, 45). Thus, ticks may outlive their hosts or live through the drastic changes in host population composition. For example, progressive feral swine invasion (60) has increased tick host diversity and abundance (61), impacting exposure to and interactions with *O. turicata* (62, 63). Consequently, overlapping *O. turicata* generations may occur. In this case, bloodmeal analysis may not accurately depict host utilization of older generation ticks that may have had exclusive access to hosts that are no longer available or diminished to younger-generation ticks.

In summary, the bloodmeal analysis techniques evaluated in this study demonstrated promising tools for determining host utilization of *O. turicata*. The DNA-based bloodmeal analysis underscored the feasibility to discern multiple-host utilization by *O. turicata* and confirmed the applicability of the *cytb* gene as a host-specific molecular marker. The SI-based bloodmeal analysis was able to distinguish host blood, *O. turicata* cohorts fed on different kinds of host blood, and nitrogen enrichment in *O. turicata* post-bloodmeal consumption, although the utility of this approach in the future may be limited to unique circumstances. Our future work is applying the DNA-based bloodmeal analysis to *O. turicata* collected in the field [(64) in review]. A comprehensive understanding of vector ecology, including host utilization, is important for studying the natural history of soft ticks, their associated tick-borne pathogens, and applications of techniques for surveillance and intervention strategies.

## Data Availability Statement

The raw data supporting the conclusions of this article will be made available by the authors, without undue reservation.

## Ethics Statement

The animal study was reviewed and approved by Institutional IACUC committee reviewed and approved the protocol.

## Author's Note

The content of this manuscript has been published in part, as a chapter in the 2017 dissertation of Hee J. Kim (65).

## Author Contributions

All authors made substantial intellectual contributions to the project including experimental design, protocol execution, assessment of results, and preparation of the manuscript.

## Conflict of Interest

The authors declare that the research was conducted in the absence of any commercial or financial relationships that could be construed as a potential conflict of interest.
